# Studies on the effectiveness of oral pellet vaccine in improving egg production and egg quality in desi chicken

**DOI:** 10.14202/vetworld.2016.900-903

**Published:** 2016-08-24

**Authors:** T. Lurthu Reetha, J. Johnson Rajeswar, T. J. Harikrishnan, K. Sukumar, P. Srinivasan, J. John Kirubakaran

**Affiliations:** 1 Tamil Nadu Veterinary and Animal Sciences University - Regional Research, Pudukkottai - 622 004, Tamil Nadu, India; 2 Department of Veterinary Microbiology, Veterinary College and Research Institute, Tirunelveli - 627 001, Tamil Nadu, India; 3 Directorate of Research, Tamil Nadu Veterinary and Animal Sciences University, Chennai - 600 051, Tamil Nadu, India; 4 Department of Veterinary Microbiology, Veterinary College and Research Institute, Namakkal - 637 002, Tamil Nadu, India; 5 Veterinary University Training and Research Centre, Nagapattinam, Tamil Nadu, India; 6 Department of Veterinary Microbiology, Madras Veterinary College, Chennai - 600 007, Tamil Nadu, India

**Keywords:** effectiveness, egg production, egg quality, desi chicken, oral pellet vaccines

## Abstract

**Aim:**

To study the effect of Newcastle disease (ND) oral pellet vaccine in egg production and egg quality in desi chicken.

**Materials and Methods:**

The study was conducted at Veterinary University Training and Research Centre, Tiruchirapalli, Tamil Nadu. A total of 48-day-old desi chicks obtained from a private hatchery in Namakkal, Tamil Nadu, were maintained under cage system of rearing up to 52 weeks of age as per standard management practices. All the 48 chicks were divided into six groups having eight chicks in each group were subjected to different treatment regimes. All the birds were challenged at 52 weeks of age with 0.5 ml dose of 10^4.0^ egg infectious dose 50 virulent ND field virus. 10 eggs from each group were randomly collected during the last 3 days of 8 weeks interval period from 28 to 52 weeks of age and were used to measure the egg quality parameters. The production performance of each group was assessed at 4 weeks interval period from 25 to 52 weeks of age.

**Results:**

In all the six treatment groups with respect to egg production, no significant difference (p≥0.05) was noticed from 25 to 52 weeks of age. Similarly, in egg weight, egg shape index and specific gravity, no significant difference (p≥0.05) was noticed from 28 to 52 weeks of age.

**Conclusion:**

From this study, it is concluded that the administration of ND oral pellet vaccine to desi chicken does not affect the egg production performance, egg weight, egg shape index, and specific gravity of egg.

## Introduction

Newcastle disease (ND) is one of the major viral diseases of poultry causing great economic losses to the poultry industry [1]. ND remains a constant threat to the poultry industry and is a limiting disease for poultry producers worldwide [2]. The native chicken varieties adopted in free-range backyard conditions contribute about 11% of total egg production in India [3]. Village poultry plays a key and often undervalued role in a rural development in many poor rural households and is a global asset for many millions who live below the poverty line [4].

A vaccination of chickens has remained as the principal method to control this disease [5,6]. Control of ND primarily consists of vaccination of flocks and culling of infected or likely infected birds [7]. An effective vaccination with conventional vaccines involves maintaining a cold chain, catching and handling individual birds, using skilled vaccinators and repeating the whole procedure sufficiently often to ensure that every bird receives at least two doses of vaccine at different times [8]. The selection of an ND vaccine for use in rural chicken will depend on the local conditions in each country. Selection criteria will include – Ease of use, cost, thermostability, immunogenicity, availability, and transportability. In circumstances where the cold chain is weak or absent, the only reliable option will be the use of thermostable ND vaccines [9].

Hence, this study was conducted with the objective to observe the effectiveness of thermostable Tamil Nadu Veterinary and Animal Sciences University (TANUVAS) oral pellet vaccine in improving egg production and egg quality in desi chicken.

## Materials and Methods

### Ethical approval

All animal procedures were performed in accordance with Institutional Animal Ethical Committee regulations (Approval No. 8/2012 of IAEC dated 10.08.2012).

### Birds and experimental design

The study was carried out at Veterinary University Training and Research Centre, Tiruchirapalli, Tamil Nadu. A total of 48-day-old desi chicks obtained from a private hatchery in Namakkal, Tamil Nadu, were maintained under cage system of rearing up to 52 weeks of age under standard management practices. All the 48 chicks were divided into six groups having eight chicks in each group. First group (T1) served as unvaccinated control, second group (T2) was primed with commercially available thermostabilized D58 vaccine followed by booster with TANUVAS oral pellet vaccine, third group (T3) was primed as well as boosted with TANUVAS oral pellet vaccine, fourth group (T4) was primed with RDV’F’ followed by booster with commercial vaccines (LaSota and R2B), fifth group (T5) was primed with commercially available thermostabilized D58 vaccine followed by booster with commercial vaccines (LaSota and R2B), sixth group (T6) was primed with TANUVAS oral pellet vaccine followed by booster with commercial vaccines (LaSota and R2B). All the birds were challenged at 52 weeks of age with 0.5 ml dose of 10^4.0^ egg infectious dose 50 (EID_50_) virulent ND field virus.

The TANUVAS oral pellet vaccine produced was standardized for safety, purity, and potency as per the specifications [10,11]. In potency test, 10 unprimed chickens aged 6 weeks old were vaccinated with oral pellet vaccine *ad libitum* and hemagglutination inhibition titer was recorded on 21 days post vaccination. If the flock average was more than 23, the batch of vaccine was considered as potent. To reconfirm this, all birds were challenged with 10^6^ EID_50_ of virulent velogenic virus obtained from Institute of Veterinary Preventive Medicine, Ranipet. All birds withstood the challenge. The different batches of vaccine produced passed this test and proved to be potent. In safety test, 10 unprimed birds were given more than minimum dose (vaccine pellets given *ad libitum*) and the birds were observed for 14 days. No local and systemic reactions were noticed. The different batches of vaccine produced passed this test and proved to be safe. In purity test, the seed was tested for bacterial and fungal contaminants and infectious bronchitis virus and found to be pure. The vaccine did not contain any aerobic or anaerobic bacteria and fungi. The vaccine was found to be potent, and the vaccinated birds withstood virulent virus challenge.

### Sample collection and analysis

The production performance of each group was assessed at 4 weeks interval period from 25 to 52 weeks of age and percent hen-day egg production was worked out. 10 eggs per treatment were randomly collected to study the physical qualities of egg like egg weight, shape index, and specific gravity were assessed by standard procedure.

### Statistical analysis

An analysis of variance with one factor was used to examine the effects of different types of vaccine on egg production, egg weight, egg shape index, and egg specific gravity. The values presented were expressed by assigned average standard error of the mean. In the case of significant difference, Tukey’s HSD test was used to separate homogeneous groups at a significant level of 5%.

## Results and Discussion

Poultry, especially chickens, are the most widely reared species worldwide and are found in great numbers [12]. Poultry production has an important economic, social and cultural benefit and plays a significant role in family nutrition in the developing countries [13]. Egg quality is the general term which refers to general standards which define both internal and external quality such as egg weight, egg length, width, egg index, shell weight, shell thickness, albumin height, albumin width, yolk height, yolk index, and haugh unit [14].

Village poultry farmers have less awareness to sort eggs for different purposes such as for hatching, marketing and home consumption purposes [15]. The egg quality could be affected by the production system among others [16]. Free range chicken production represents an important system for providing high-quality protein and income to the growing human population especially for the women [17]. Due to the low production (annual egg production: 50-60 nos.) nature of desi birds, their contribution to the total egg output was almost static for the last few decades [18].

The thermostable vaccine induces protective immunity among free-range chickens when correctly applied. It is cheaper and thus makes it affordable to all farmers to use and it is easy to administer by farmers. It contributes to the improvement of household food security in many developing countries the control of ND will contribute to improved village poultry production. The thermostable vaccine of ND-I 2 and V4 (Australian strains) is used to control ND in village chickens in Africa and Asia [19].

In this study with respect to egg production performance, no significant difference was noticed up to 52 weeks of age (p≥0.05). The overall mean of percent egg production in T2 and T6 is similar and also T1, T3 and T5 are similar followed by T4 (Table-1). In earlier study, similar finding was reported [20] that average annual egg production of the native chicken was 30-60 eggs under village conditions and that this could be improved to 80-100 eggs on-station by proper vaccination.

**Table-1:** Effect of different ND vaccine and vaccination regimen in desi chicken on percent hen day egg production.

Treatment	Mean egg production							Overall mean	Average egg number per bird
	
	25-28 weeks	29-32 weeks	33-36 weeks	37-40 weeks	41-44 weeks	45-48 weeks	49-52 weeks	Percent egg production	
T1 - Control	12.50	19.64	23.21	21.43	20.54	19.64	18.75	18.75	36.75
T2 - D58 and oral pellet vaccine	13.39	19.64	22.32	21.43	20.54	20.54	19.64	19.64	38.50
T3 - Oral pellet vaccine	14.29	20.54	24.11	22.32	20.54	19.64	18.75	18.75	36.75
T4 - Commercial vaccine	14.29	19.64	23.21	21.43	19.64	18.75	17.86	17.86	35.00
T5 - D58 and commercial vaccine	14.29	19.64	22.32	21.43	20.54	19.64	18.75	18.75	36.75
T6 - Oral pellet vaccine and commercial vaccine	14.29	19.64	22.32	21.43	20.54	20.54	19.64	19.64	38.50
p value	0.16								

ND: Newcastle disease

In all the six treatment groups with respect to egg weight, no significant difference was noticed from 28 to 36 weeks of age, 37 to 44 weeks of age, and 45 to 52 weeks of age (p≥0.05). The mean egg weight in 45-52 weeks of age was maximum followed by 37-44 weeks of age and 28-36 weeks of age (Table-2). The mean egg weight of 38 g and 40.73 g was reported in Fulani-ecotype native chicken eggs [21].

**Table-2:** Effect of different ND vaccine and vaccination regimen in desi chicken on egg weight (g) (mean±SE).

Treatment	Mean egg weight (g)		

	28-36 weeks	37-44 weeks	45-52 weeks
T1 - Control	38.22±0.11	38.86±0.15	39.88±0.17
T2 - D58 and oral pellet vaccine	38.12±0.19	38.87±0.18	39.87±0.19
T3 - Oral pellet vaccine	38.25±0.18	38.84±0.16	39.94±0.17
T4 - Commercial vaccine	38.07±0.17	38.98±0.18	39.98±0.20
T5 - D58 and commercial vaccine	38.04±0.17	38.84±0.17	40.01±0.19
T6 - Oral pellet vaccine and commercial vaccine	37.94±0.12	38.94±0.15	40.05±0.20
p value	0.75	0.97	0.82

Each value is a mean of 10 observations. SE: Standard error, ND: Newcastle disease

In this study with respect to egg shape index, no significant difference was noticed between different treatment groups from 28 to 36 weeks of age, 37 to 44 weeks of age, and 45 to 52 weeks of age (p≥0.05). The mean egg shape index of all treatment groups ranged from 77.14±0.23 to 77.83±0.10 during 28 to 52 weeks of age (Table-3). In another recent study [22] reported that the egg shape index of naked neck birds as 77.51±1.80.

**Table-3:** Effect of different ND vaccine and vaccination regimen in desi chicken on egg shape index (mean±SE).

Treatment	Mean egg shape index		

	28-36 weeks	37-44 weeks	45-52 weeks
T1 - Control	77.61±0.08	77.14±0.23	77.38±0.21
T2 - D58 and oral pellet vaccine	77.32±0.20	77.66±0.15	77.78±0.16
T3 - Oral pellet vaccine	77.43±0.22	77.41±0.17	77.83±0.10
T4 - Commercial vaccine	77.71±0.06	77.57±0.14	77.65±0.18
T5 - D58 and commercial vaccine	77.72±0.11	77.29±0.21	77.37±0.10
T6 - Oral pellet vaccine and commercial vaccine	77.66±0.09	77.58±0.14	77.47±0.12
p value	0.18	0.36	0.16

Each value is a mean of 10 observations. SE: Standard error, ND: Newcastle disease

In this study with respect to specific gravity of egg, no significant difference was noticed from 28 to 36 weeks of age, 37 to 44 weeks of age, and 45 to 52 weeks of age (p≥0.05). The mean specific gravity of all treatment groups ranged from 1.130±0.009 to 1.186±0.023 during 28 to 52 weeks of age (Table-4). The external and internal egg quality traits of local hill fowl in Uttarakhand under an intensive system of rearing and recorded that the specific gravity as 1.08 [23].

**Table-4:** Effect of different ND vaccine and vaccination regimen in desi chicken on egg specific gravity (mean±SE).

Treatment	Mean specific gravity		

	28-36 weeks	37-44 weeks	45-52 weeks
T1 - Control	1.131±0.004	1.158±0.007	1.164±0.008
T2 - D58 and oral pellet vaccine	1.130±0.009	1.165±0.005	1.164±0.004
T3 - Oral pellet vaccine	1.150±0.014	1.163±0.006	1.131±0.019
T4 - Commercial vaccine	1.174±0.024	1.145±0.004	1.162±0.006
T5 - D58 and commercial vaccine	1.149±0.008	1.152±0.008	1.161±0.008
T6 - Oral pellet vaccine and commercial vaccine	1.186±0.023	1.144±0.006	1.142±0.012
p value	0.115	0.078	0.162

Each value is a mean of 10 observations. SE: Standard error, ND: Newcastle disease

The challenged birds were observed 10 days for the development of clinical signs, mortality, and lesions. No mortality was observed in any of the vaccinated group whereas 100% mortality was recorded in the unvaccinated control group during the observation period. The challenged control birds exhibited clinical signs like greenish white diarrhea and torticollis. Post mortem examination revealed petechial hemorrhages on the tip of proventriculus papillae.

## Conclusion

From this study, it is concluded that the administration of ND oral pellet vaccine to desi chicken does not affect the egg production performance and other egg quality traits. The thermostable TANUVAS oral pellet vaccine had found to be more advantageous, and the vaccine can support poultry farmers to get rid from ND globally.

## Authors’ Contributions

This study was a part of TLR’s original research work during Ph.D. thesis program. JJR, TJH and KS had designed the plan of work. PS helped the laboratory work. JJK tested the safety of the vaccine. TLR, JJ, TJH, KS, PS and JJK analyzed the results. All the authors read and approved the final manuscript.
